# Monosymmetros Cephalothoracopagus Tetrabrachius and Tetrapus Piglets with Syndromic Evolution

**DOI:** 10.3390/ani14142127

**Published:** 2024-07-21

**Authors:** Simona Marc, Ioan Claudiu Crăciun, Bogdan Sicoe, Jelena Savici, Oana Maria Boldura, Cristina Paul, Gabriel Otavă, Cristina Văduva, Adrian Stancu

**Affiliations:** 1 Faculty of Veterinary Medicine, University of Life Sciences “King Mihai I” from Timișoara, Calea Aradului 119, 300645 Timișoara, Romania; simona.marc@usvt.ro (S.M.); craciunionutz86@yahoo.com (I.C.C.); bogdan.sicoe@usvt.ro (B.S.); jelenasavici@usvt.ro (J.S.); oanaboldura@usvt.ro (O.M.B.); gabrielotava@usvt.ro (G.O.); cristina.vaduva@usvt.ro (C.V.); adrianstancu@usvt.ro (A.S.); 2 Department of Applied Chemistry and Engineering of Organic and Natural Compounds, Faculty of Industrial Chemistry and Environmental Engineering, Politehnica University Timișoara, Vasile Pârvan 6, 300223 Timișoara, Romania

**Keywords:** conjoined twins, swine congenital malformations, anencephaly, palatoschisis, embryogenesis

## Abstract

**Simple Summary:**

Simple Summary: This article focuses on conjoined twins in mammals, the theories that account for their development, their various clinical presentations, and the possible molecular implications of their development. We also present two different cases of monocephalic thoracopagus conjoined twin piglets. In both cases, the piglets also had other congenital malformations. The cases are described using necropsy and computed tomography scanning.

**Abstract:**

Conjoined twins are rare congenital malformations that have been reported in mammals. Two different cases are presented in this study. Case No. 1 features monocephalic, thoracopagus-conjoined twin piglets with anencephaly and palatoschisis of the Pietrain breed, and case No. 2 features monocephalic, thoracopagus conjoined twin piglets with palatoschisis and bifid root tongue of a mixed breed. These cases were examined using post-mortem and computed tomography (CT) examinations. In both cases, the conjoined symmetrical twins had a single head, one neck, and fused thoracic cavities, while the abdominal cavities were separated. Similarly, in both cases, they had four forelimbs and four hindlimbs and duplicated foramen magnum. During CT examination, in case No. 1, severe abnormalities were observed in the skull and vertebral column. In the left twin, occult dysraphism was seen from the C2 vertebra until the end of the vertebral column, and in the right twin, from the C3 vertebra until the end of the state vertebral level. In case No. 2, the oral cavity contained a tongue with a bifid root connected with one hyoid bone, and the soft palate presented a small cleft. During CT examination, the parietal bone and the occipital bones were partially duplicated. This case also presented occult dysraphism, but only in the cervical vertebrae, C1–C6 for the left twin and C1–C5 for the right twin. In both cases, abnormalities of the internal organs were revealed during necropsy. Conjoined twins with multiple congenital anomalies presented here enhance our understanding of the various clinical forms of conjoined cases in veterinary medicine.

## 1. Introduction

Conjoined twins represent one of the most interesting, rare congenital malformations seen in mammals. They have been reported in cattle [1,2,3,4], buffaloes [5], sheep [5,6,7,8,9], goats [10], pigs [11,12], dogs [13], cats [14], wild animals [15,16], laboratory animals [17], guinea pigs [18], crested geckos (*Correlophus ciliatus*) [19], and fish [20]. Throughout history (1671–2006), 19 cases of conjoined twins have been reported [15]. In laboratory animals, cases are also rare: 1 in 10,000 for rats and 1 in 4000 for rabbits [17]. Conjoined twins also probably occur in other species but have not been reported in the scientific literature. 

The mechanism behind the development of conjoined twins is not exactly known, but there are two main theories: fission versus fusion. *Fission theory* proposes a failure in the formation of one or more constituents of the body during embryonic development after a division of the embryonic disc later than the 12–13th-day post-fertilization [21,22]. The types of twins, depending on the time of embryo splitting, can be: type I monozygous dichorionic–diamniotic twins (18–36%), where the splitting of the embryo takes place on day 1–3 (early embryo, morula stage); type II monozygous monochorionic–diamniotic twins, where the splitting of the embryo takes place on day 4–8, and two embryoblasts form a single blastocyst without interfering with the trophoblast (in the blastocyst stage, cell differentiation take place) (80%); *type III* monozygous monochorionic–monoamniotic twins, where the splitting of the embryo takes place on day 8–12, shortly before or during the formation of the primitive streak (late blastocyste stage) (2–4%) [22,23]. 

The second theory is *fusion theory* (in the case of dizygotic twins or not), which suggests that initially, two separate monoovular embryonic discs fuse together, and the embryos are blocked or subsequently joined by cell adhesion molecules in the fetus. The author suggested that the inner cell mass divides into two separate monozygotic embryonic primordia that share either the amniotic cavity or the yolk sac [24]. They may come in contact and reunite, resulting in ventrally, laterally, caudally, or dorsally conjoined twins [24,25]. Some researchers no longer consider conjoined twins to be the result of the fusion of monozygotic twins [21]. Spencer also proposed a theoretical model, “spherical theory”, through which the two embryonic disks do not lie adjacent to each other but “float” on the outer surface of a spherical yolk sac, always resulting in homologous ventrally, caudally, or laterally conjoined twins [24]. 

Even so, the heterokaryotype cellular mosaicism does not accurately indicate dizygosity because postzygotic mitotic mutations can occur. In humans, monochorionic biamniotic conjoined twins, a heteropagus type with discordant sex chromosomes, non-mosaic chromosomal discordance, and no additional pathogenic copy number variants, were identified in both biopsies. The zygosity test confirmed monozygosity [26].

These two theories cannot be applied in all cases [25]. The mono- or bivitelline origin of conjoined twins can be demonstrated through genetic analysis. An example is the case of a German Holstein calf with caudal duplication associated with polydactyly, a bifid scrotum, a diphallus, and atresia ani with recto-vesical fistula that was demonstrated by using SNP50 v2 BeadChip to be monozygotic [27]. 

There are two important classifications of conjoined twins. The first one is based on the orientation of the fusion. They can be symmetrical twins (diplopagus) if they have parallel cranio–caudal axes or asymmetrical twins (heteropagus or parasitic twin) if the orientation of an incomplete twin (parasitic twin) is perpendicular to a normally developed twin (autosite). The parasitic twin can be attached at any part of the autosite, usually in the hypogastric or suprapubic region. In heteropagus phenotypes, the autosite retains parts of the parasitic twin that have remained vascularized after its death. The etiology of the embryonic death of the parasitic twin is thought to be caused by ischemic atrophy or insufficient cardiac function [28]. A rare case of pygopagus tetrapus parasitic twin was identified in a human case with positive surgical intervention [29]. Heteropagus twins have a generally favorable prognosis. The formation of teratoma at the site of retained parasite tissue can be a complication after heteropagus surgical intervention [28]. 

The other classification is based on the anatomical region where the twins are joined: cephalopagus (fused ventrally at the level of the head and chest), thoracopagus (fused ventrally at the level of the chest), omphalopagus (fused ventrally at the level of the abdomen), ischiopagus (united ventrally at the level of the pelvis), pygopagus (fused caudally, at the sacrum, coccyx, or perineum), rachipagus (fused dorsally at the spine), diprosopus (one body with two faces), or dicephalus (one body, two heads) [24]. 

Another classification of the conjunction is the non-dorsal or dorsal union. Non-dorsal conjunction is divided into ventral, lateral, and caudal conjunction types. All non-dorsal conjoined twins have a single umbilical cord, and all dorsal conjoined twins have two umbilical cords and individual internal organs [30]. For the dorsal conjunction type, the proposed cause is the secondary fusion of two initially separate monozygotic twins [25]. 

Based on arguments proposed by Boer et al. [25], incomplete fission cannot be the cause of non-dorsally conjoined twins. The authors showed that all non-dorsally conjoined twins are affected by the same neo-axial orientation and/or interaction aplasia. Neo-axial orientation refers to the mechanism by which opposing homologous structures are divided in the median plane, after which the two halves will form laterally. Interaction aplasia appears when two primordia lines have a parallel position and, due to possible aberrant concentrations of morphogenes, one of the primordia fails to develop.

The clinical forms are very varied; for example, in the case of cephalopagus twins, depending on the variability of the degree of the rostral union of the embryonic discs, they are classified as symmetric ventral union (classical cephalopagus), where, clinically, they present two faces. Next is moderate asymmetry union, which is clinical with a diminished posterior face and brain, variable mouth, and eyes, nose, and ears present. Following is severe asymmetry with the complete absence of the posterior face (as described by Kompanje et al. for conjoined leopard twins) [15]. Another type is extreme asymmetry, resulting in anencephaly of the conjoined brain, either with one face or with two laterally united faces on the same side but with the ventral union of the body down to the umbilicus. Last is maximal asymmetry, resulting in prosopothoracopagus with separate brains but with a ventral union of the trunks [24].

Atypical twins have also been reported in humans, such as chimeric twins, where a single offspring contains two populations of different genetic cell origination from two different zygotes, described in monochorionic dizygotic placentation. Next are mirror-image twins in asymmetrical monozygotic twins from late zygotic splitting at 9–12 days just prior to the formation of conjoined twins. There are also polar body twins, complete hydatidiform mole, vanishing twins, fetus papyraceus, enclosed fetus in a fetus, internal teratoma, and acardic connected via the placenta [22,29].

These fascinating cases of conjoined twins have been investigated throughout the history of human and veterinary medicine. Descriptions of cases can be seen in preclinical disciplines (e.g., anatomy, embryology, genetics) or older books, but recently, conjoined twin cases reported in veterinary medicine are still low. To enhance our knowledge about the various types of conjoined twins in mammals, we review current literature data on conjoined twins and provide a post-mortem description, computed tomography scanning, and necropsy findings in two cases of male conjoined twin piglets.

## 2. Materials and Methods

### 2.1. Animal 

In case No. 1, the stillborn male conjoined piglets described here, along with six other piglets, were the offspring of a one-year-old primiparous Pietrain sow. The piglets were born on 21 May 2023, on a farm. The other six piglets (four males and two females) had no congenital abnormalities and were alive. The parturition was natural, with no other problems. There was no history of previous congenital malformations in piglets originating from the same boar on the farm. 

In case No. 2, the stillborn male conjoined piglets described, along with nine other piglets (four males and five females), were the offspring of a multiparous mixed-breed Pietrain/Large White sow at her third parturition. The piglets were born on 14 February 2024, on a farm. The other nine piglets had no congenital abnormalities and were alive. The parturition was natural, with no other problems. There was no history of previous congenital malformations in piglets originating from the same parents on the farm. Due to the rare incidence of conjoined twins, both cases were submitted to the University of Life Sciences “King Mihai I” from Timișoara, the Faculty of Veterinary Medicine, to the genetics discipline for didactic and research purposes.

### 2.2. Computed Tomography Investigation

The acquisition of CT (computed tomography) scan images was performed with the body positioned in a lateral recumbency. CT scans were performed with a Siemens Somatom Definition AS 64 scanner (Siemens Healthineers, Erlangen, Germany), using conventional settings (90 kV, 100 mAs) and a slice thickness of 0.6 mm.

The computer tomography images that were in the form of VRT Imagines (Volume Rending Technology) and MPR Imagines (Multiplanar reconstruction images) were analyzed using the CT’s own DICOM viewer, as well as a MicroDICOM viewer.

### 2.3. Necropsy Investigation

After post-mortem and CT investigations, the specimen was prepared to investigate specific internal organs. It was fixed in 9% non-buffered formalin, and after a period of time, internal organs were examined. 

## 3. Results

### 3.1. Post Mortem Description—Case No. 1 

**Figure 1:**
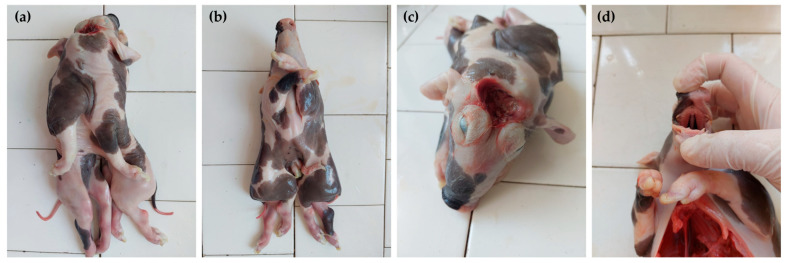
The clinical aspects of the conjoined twins (case No. 1). Dorsal view (**a**), ventral view (**b**), rostral view of the head (**c**), and cleft palate view (**d**).

The conjoined symmetrical twins had a single head and neck, two thoracic cavities fused together, and two separated abdominal cavities. The conjoined stillborn piglets displayed distinctive signs of exencephaly–anencephaly with a reddish membrane that enveloped the underdeveloped open part of the cranial cavity. The shallow orbits gave rise to ocular protrusion. The two external ears were normal. The oral cavity contained one tongue connected with one hyoid bone. The hard palate presented a complete cleft. The paired nasal cavities were visible through the cleft. The piglets had four forelimbs and four hindlimbs. All limbs were developed normally. Caudally to the single umbilicus, the bodies were completely separated (Figure 1). 

During post-mortem investigations, the newborn was weighed, and the main body measurements were carried out (Table 1). 

### 3.2. Post Mortem Description—Case No. 2 

**Figure 2:**
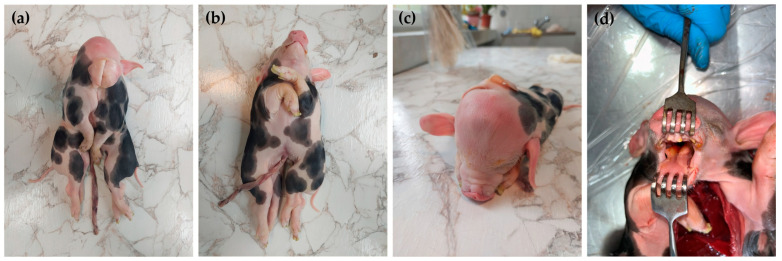
The clinical aspects of the conjoined twins (case No. 2). Dorsal view (**a**), ventral view (**b**), rostral view of the head (**c**), and bifid root tongue view (**d**).

The conjoined symmetrical twins had a single head, two normal external ears, two abnormal median ears, one neck, two thoracic cavities fused together, and two separated abdominal cavities. The lateral external ears of each piglet were normal, and those of the median/fused part were rudimentary. The oral cavity contained one tongue with a bifid root connected with one hyoid bone. The soft palate presented a small cleft. The piglets had four forelimbs and four hindlimbs. All limbs were developed normally. Caudally to the single umbilicus, the bodies were completely separated (Figure 2). 

During post-mortem investigations, the twins were weighed, and the main body measurements were performed (Table 2).

### 3.3. CT Examination in Case No. 1

**Figure 3:**
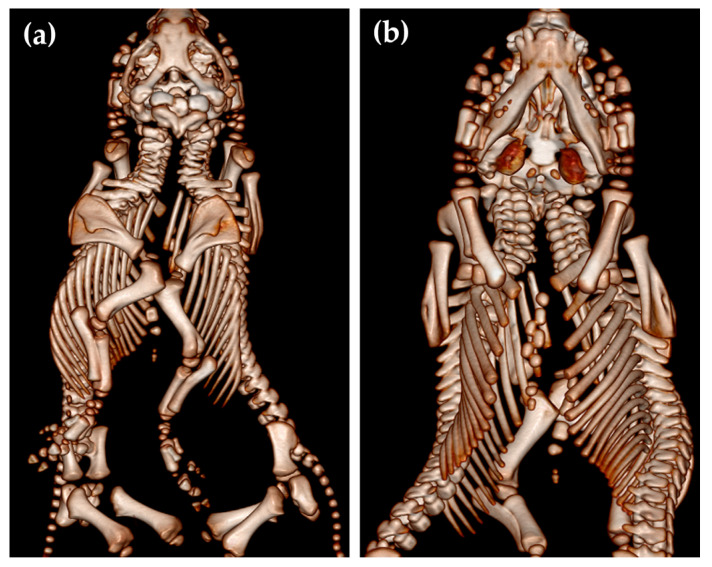
Dorsal (**a**) and ventral (**b**) view of the conjoined twins (case No. 1).

As usual, these cephalopagus conjoined twins had two separate vertebral columns with two foramina magna of the skull (Figure 3). 

**Figure 4:**
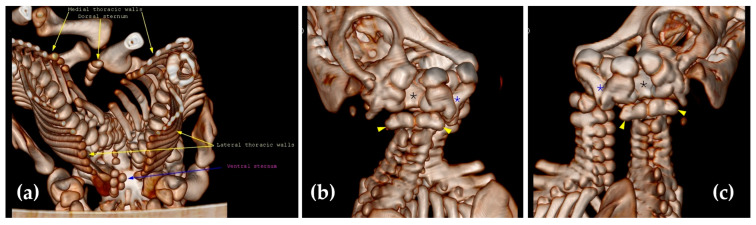
CT scan of the thoracal cavities’ fusion (**a**). The right paracondylar process of the left twin fused with the left paracondylar process of the right twin (blue asterisk); there are two foramina magna (black asterisk), and the atlas is also indicated (yellow arrows). The squamous part of the occipital bone was duplicated and incompletely formed; a partial lack of parietal bone is seen in (**b**,**c**) (case No. 1).

During the CT examination of case No. 1, the caudal part of the frontal bone and the parietal bone were found to be absent, with no sagittal crest of the parietal bone. The occipital bone was without external occipital protuberance, while the squamous part of the occipital bone was duplicated. The temporal bones were missing. The foramen magnum was duplicated, while the occipital bone presented four occipital condyles. The right paracondylar process of the occipital bone of the left twin was fused with the left paracondylar process of the occipital bone of the right twin (Figure 4b,c).

Two shared sternums existed in the thoracic cavity of conjoined twins; each twin had its own set of ribs. The two sternums presented normal sternebrae with normal xiphoid processes. The dorsal sternum merged with the left thoracic wall from the body of the right twin and with the right thoracic wall from the body of the left twin, while the ventral sternum fused with the left thoracic wall from the body of the left twin and with the thoracic wall from the body of the right twin (Figure 4a). 

The other skull bone structures, such as the nasal bone, incisive bone, frontal bone, zygomatic process of the frontal bone, temporal bone, the zygomatic process of the temporal bone, temporal bone zygomatic bone left and right zygomatic bone temporal process, developed normally. The complete CT examination of the left twin’s spine revealed that the dorsal vertebral arch of C2–C7 and the dorsal vertebral arch of all the thoracic, lumbar, and coccygeal vertebras were opened. In the right twin, the dorsal vertebral arch of C3–C7 and all the thoracic, lumbar, and coccygeal vertebrae were opened.

### 3.4. CT Examination in Case No. 2

**Figure 5:**
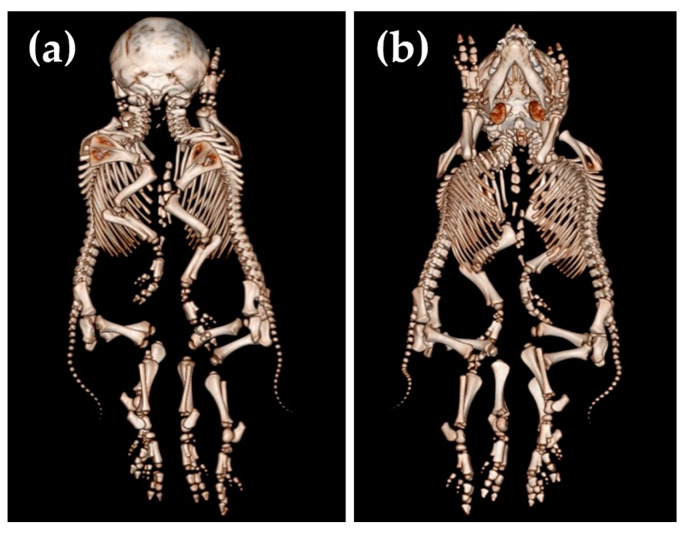
Dorsal (**a**) and ventral (**b**) view of the conjoined twins, case No. 2.

The complete CT examination of the left twin’s spine revealed that the dorsal vertebral arch of C1–C6 was opened. In the right piglet, the dorsal neural arch of the C1–C5 vertebrae was opened, while all the thoracal, lumbar, and coccyx vertebrae were closed in both left and right twins (Figure 5).

**Figure 6:**
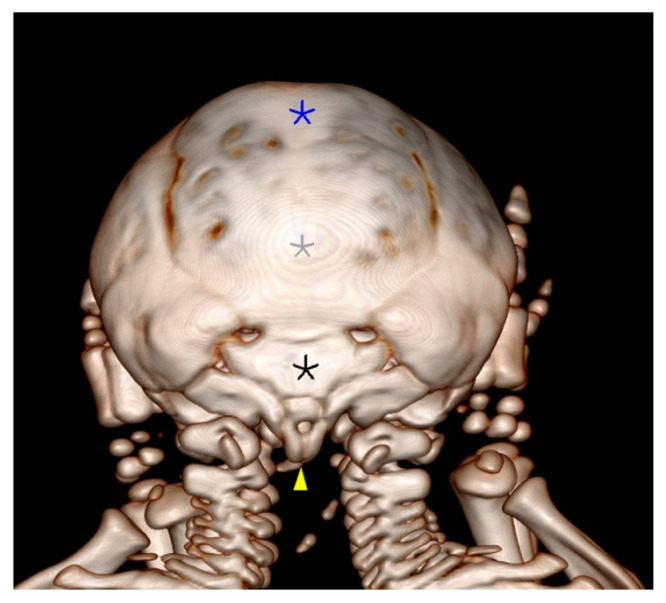
Caudal view of the skull (case No. 2), where the blue asterisk represents the fusion between the right frontal bone of the left twin and the left frontal bone of the right twin, the grey asterisk represents the fusion between the right parietal bone of the left twin and the left parietal bone of the right twin, the black asterisk represents the fusion between the right temporal bone of the left twin and the left temporal bone of the right twin, and the yellow arrowhead represents the fusion between the right paracondylar process of the occipital bone of the left twin and the left paracondylar process of the occipital bone of the right twin.

During the CT examination in case No. 2, the parietal bone and the occipital bones were found to be duplicated. The occipital bone was without external occipital protuberance. The right paracondylar process of the occipital bone of the left twin fused with the left paracondylar process of the occipital bone of the right twin. The two holes visible on CT are the consequence of temporal bone fusion (Figure 6).

**Figure 7:**
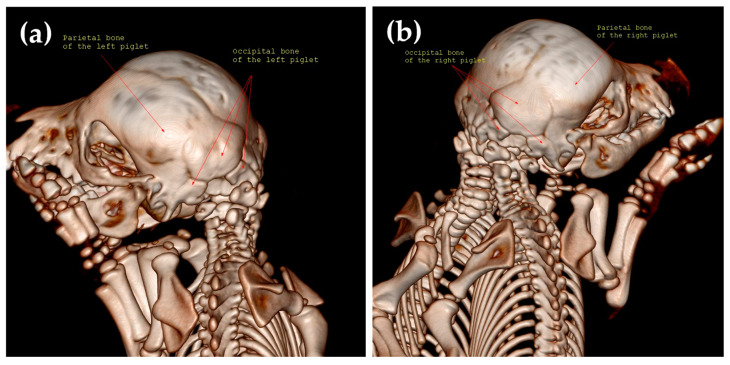
The caudo-lateral view of the left twin (**a**) and the right twin (**b**).

In case No. 2, the skull size increased due to partial duplication of the frontal, parietal, temporal, and occipital bones. Further, the conjoined twins had four ears, but the medial ones were rudimentary, and only the pinna auricularis was identified. The foramen magnum was duplicated (Figure 7). 

The other bone structures of the skull, such as the nasal bone, the incisive bone, the zygomatic process of the frontal bone, the temporal bone, the zygomatic process of the temporal bone, and the left and right temporal process of the zygomatic bone, were normally developed.

### 3.5. Necropsy Examination in Case No. 1

**Figure 8:**
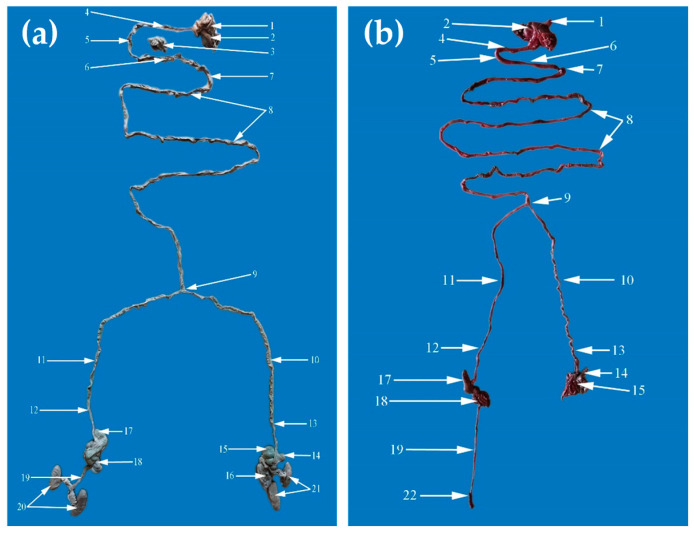
The gastrointestinal tract of the conjoined twins, case No. 1 (**a**) and case No. 2 (**b**). **1.** Esophagus; **2.** Stomach; **3.** Pancreas; **4.** Descending duodenum; **5.** Transverse duodenum; **6.** Ascending duodenum; **7.** Duodenojejunal flexure; **8.** Jejunum; **9.** Furcation of the jejunum into the left and right jejunum for the correspondent body; **10.** Left terminal jejunum; **11.** Right terminal jejunum; **12.** Right ileum; **13.** Left ileum; **14.** Left cecum; **15.** Left ascending colon; **16.** Left descending colon; **17.** Right cecum; **18.** Right ascending colon; **19.** Right descending colon; **20.** Right kidneys; **21.** Left kidneys; **22.** Right rectum.

In case No. 1, the dissection of the conjoined twins revealed the arrangement of the internal organs. The internal conformation of the heart was normal. The configuration of the left and right ventricle was regular. They had two tracheas with two pairs of lungs and presented two thoracic aorta and two abdominal aortas. The thoracic cavity was separated from the abdominal cavity by a normal diaphragm. The gastrointestinal segments were common until the jejunum, and after that point, the rest of the gastrointestinal tract was divided. Therefore, the conjoined twins had a single tongue, one larynx, one esophagus, one stomach, and one duodenum, and at one point, the jejunum was separated into two parts. From that part, the piglets had two of the following, left and right: terminal jejunum, ileum, cecum, ascending colon, and descending colon, which ended with two anuses, normally developed. The twins’ liver was large and multilobed, typical for the swine species, with one gallbladder. The urogenital apparatus was separate for each piglet (Figure 8). The testicles had descended into the scrotum. The extrapelvic urethra was normally opened in the middle ventral abdominal region. 

### 3.6. Necropsy Examination in Case No. 2

**Figure 9:**
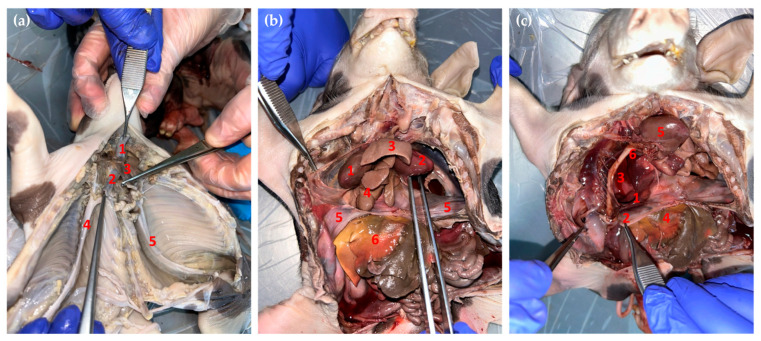
Necropsy of the conjoined twins. (**a**) Caudoventral aspect of the thoracic cavity (case No. 1): **1.** Dorsal trachea; **2.** Ventral trachea; **3.** Esophagus; **4.** Thoracic aorta from right body; **5.** Thoracic aorta from left body. (**b**) Ventral view of the thoracic and abdominal cavities of the conjoined twins (case No. 2): **1.** Right heart; **2.** Left heart; **3.** Lungs from the left body; **4.** Lungs from the right body; **5.** Diaphragm; **6.** The liver of the right body. (**c**) Ventral view of the thoracic and abdominal cavities of the conjoined twins (case No. 2): **1.** Diaphragmatic fissure; **2.** Diaphragm; **3.** The liver from the left body herniated into the thoracic cavities through the diaphragm; **4.** Right liver; **5.** Right heart; **6.** Right thoracic aorta.

In case No. 2, the necropsy of the conjoined twins revealed the arrangement of the internal organs (Figure 9). The piglets had two unequal hearts. The right heart was larger than the left one, and the internal and external conformation of the hearts was similar to those of normal piglets. It presented two aortas; both emerged from the left ventricle but with different hearts and ran craniolateral in opposite directions. They intersected in a median plane, and they poured into each other. At this level, they had many branches, which formed the brachiocephalic trunk. Then, they ran caudo-dorsally for each body, formed the aortic arch attached to the vertebral column, and, on this level, started the thoracic aorta. The thoracic aorta provides many pare branches dorsally, which form the intercostal artery. These arteries supply the thoracic wall; then, the thoracic aorta passes by the diaphragm through the aortic ostium. The abdominal aorta, through its branches, supplies the abdominal and pelvic cavities, the internal organs, and the pelvic limbs. The cranial vena cava receives blood from the head, neck, forelimbs, and partially the thoracic wall and transmits to the right atrium of the right heart.

The conjoined twins had two larynxes, two tracheas, and two pairs of lungs. The esophagus was located between the tracheas and passed through the diaphragm of the left body. The diaphragm had portions that adhered to the liver. In the right twin, the left half of the diaphragm was incomplete, causing herniation of the caudate lobe of the left liver, together with part of the gastrointestinal mass. The left liver had six lobes and presented a gallbladder. The quadrate lobe and the medial right lobe of the left liver were crossed by the umbilical vein. The caudate lobe was highly developed. The left liver was arranged transversely to reach the body of the right piglet. The right liver had six lobes (right lateral, medial, quadrate, caudate, left lateral, and left medial lobe), the gallbladder was missing, and it was crossed by the umbilical vein. The conjoined twins presented one spleen and one stomach. The stomach and spleen belonged to the left twin. The gastrointestinal segments were similar to those of case No. 1. 

## 4. Discussion

There are no recent reports on the incidence of conjoined piglets, but older reports indicate a rate of 0.6%, and 36.8% of them had a cleft palate (21 out of 57 piglets) [31]. Another study estimated that the conjoined twinning rate for all swine births is 0.048% [32].

Conjoined twins usually have a syndromic evolution with different organs and systems affected, not only the conjoined segments [33]. They can share anomalies such as duplications, hypoplasia, and/or aplasia of the limbs, cardiac duplications, cardiac hypoplasia, etc. [34]. 

In case No. 1, a neural tube defect (NTD), exencephaly–anencephaly, was observed. NTD, such as anencephaly, was reported in diprosopus parapagus human twins and appears to be related to notochordal angulation and interaction aplasia of the medial parts of the two neural grooves that are almost absent. The remaining parts of the neural folds are so close that the closure of the neural tube is affected [34]. A similar case of cephalothoracopagus twins with anencephaly and palatoschisis was described by Kulawik et al. [33] in a Polish Large White breed. This lethal neural tube defect is caused by the lack of fusion of the cephalic folds during primary neurulation, which will cause the absence of ectodermal tissue that should form the skeletal and muscular structures that cover the underlying neural structures. Due to this maldevelopment, the skull is partially missing, and the neural structures remain open, defined as exencephaly. Although the brainstem, cerebellum, and spinal cord are present, only part of the diencephalon can be preserved [35,36,37]. Anencephaly can be observed due to degeneration and neural deficits caused by the exposure of the brain tissue to the amniotic fluid. This neural tube defect is rarely reported in domestic animals [38,39,40]. The etiology of anencephaly is supposed to have a multifactorial pattern because genetic factors or/and environmental factors are incriminated [41,42]. 

The two cases presented here had two vertebral columns, starting from the cervical ones with two sternums. Similar findings were reported by Kulawik et al. in piglets [33] and Kompanje et al. in leopards [15], but different observations were made by McManus et al. [11], wherein the piglets had separate vertebral columns caudally from the cervical region. 

Both cases revealed occult dysraphism during the CT scanning. In the first case, the dorsal vertebral arch was open in the left twin from C2 to the end of the vertebral column, while in the right twin, it was open from C3 to the end of the vertebral column. In the other case, occult dysraphism was observed only in the cervical region, C1–C6 for the left twin and C1–C5 for the right twin. Normally, the vertebral arch is closed due to the chondrification centers that appear within the mesenchymal template, which enclose the notochord and developing neural tube, and it is followed by ossification [43]. The failure of the dorsal neural arch to close is perhaps due to no migration of the sclerotomes around the dorsal part of the neural tube and with no chondrification and ossification follow-up.

In both of our cases of conjoined twins, they presented severe palatoschisis, being the only malformation in the oral cavity for case No. 1. However, in case No. 2, a double root tongue was also observed. 

Palatoschisis or cleft palate (CP) (OMIA 000197-9685) is a developmental defect of the palate that results from a failure of the medial fusion of the horizontal plates of the palatine bones and manifests itself as a separation of the hard or/and soft palate. It has been reported in cattle [44,45], dogs, especially brachycephalic breeds [46], horses [47], and cats [40,48]. The palate malformation in animals presents a range of signs, from a small pinhole cleft to a complete hard palate deficit. In this study, the cleft was complete in case No. 1 and partial in case No. 2. Palatoschisis was also seen in monocephalus tetrabrachius and tetrapus conjoined cases [49] and in a monozygotic diprosopiasis Holstein calf [3].

In case No. 2, a double root tongue was observed, a clinical form of bifid tongue, cleft tongue, or glossoschisis. This congenital defect probably has many causes, such as genetic factors that cause the failure of mesodermal migration into the midline structures of the mandibular portion of the first brachial arch; amniotic constriction bands in the region of the first brachial arch, intense genetic improvement, including inbreeding [50], or environmental factors [51]. A bifid tongue was identified in other cases of conjoined twin piglets [33].

The genito-urinary apparatuses were normal for each piglet conjoined in both cases and similar to others [1,33]. Cases with ambiguous internal reproductive segments, such as one testis and one lateral kidney [11] in diprosopus–dipygus conjoined twin piglets with one pair of forelimbs and two pairs of hindlimbs, were reported. A dipygus with an asymmetric conjoined twin in a male Holstein calf with two extra limbs in the pelvic region and a single internal abnormality with one extra kidney was reported [2]. In dogs, several cases of monocephalus dipygus puppies have been reported [13]. In one such case, the puppy had symmetrical caudal duplication (dipygus) in the lumbosacral region (rachipagus), with complete duplication of the urogenital system and duplication of the large intestines caudal from the ileum [13]. 

The gastrointestinal segments were similar between the cases, with some exceptions. In both cases, from the jejunum to the end of the gastrointestinal tractus, the segments were duplicated, with two left and right terminal jejunum, ileum, cecum, ascending colon, and descending colon that ended with two anuses. In case No. 1, the conjoined piglets had a large, multilobed liver, typical for swine species, with a gallbladder. In case No. 2, the twins presented with two livers; the caudate lobe of the left liver was herniated through the diaphragm, and the right liver had no gallbladder. A similar duplication of the digestive system from the jejunum was seen in conjoined piglets [33]. An interesting case was reported in bovines, where a conjoined twin Holstein heifer [1] was dicephalus and tetrabrachius fused in the thoracic region with two separate vertebral columns, a single pelvis, and only two hindlimbs. The internal organs were doubled, with two hearts, two sets of lungs, two esophagi, and two stomachs, but caudal from the pyloric sphincters, and all the digestive and urogenital systems were single, with a single anus. Based on anatomical aspects, the possible cause may be fission followed by the fusion of parallel embryonic axes.

Every case of conjoined twins has its own specific internal characteristics. However, external features of conjoined twins reported in veterinary medicine tend to be more prevalent with caudal duplication (dypygus) in sheep and pigs and with cranial duplication (diprosopus) in cattle [52]. Duplication of both the cranial and caudal axes can occur [11]. Regarding the sex of the conjoined twin, a predominance of female cases was noticed (70%), of which 40% were stillborn [29,53].

The molecular mechanism with possible implications in conjoined twins is related to the duplication of the primitive streak and the very early asymmetry pattern of the embryo. Physiologically, the hypoblast is known to control the cell movement from the epiblast, followed by the primitive streak and the appearance of bilateral symmetry [25]. In mice, the hypoblast is essential for the correct positioning of the primitive streak, demonstrated through knockout hypoblast mouse embryo cells, which leads to an ectopic primitive streak or its duplication [25]. 

The position of the two primitive streaks will influence the signaling molecules responsible for symmetry. Thus, in the craniopagus and ischiopagus twins, the two primitive streaks are end to end, and the signaling molecules of one side will not affect the other side. However, in dicephalus and thoracopagus twins, the primitive streaks are adjacent, and molecule cross-signaling is possible, which can induce laterality defects, such as orofacial clefts and anencephaly [30,54,55]. 

Primitive streak formation is based on a network of mainly three cascade signaling pathways: *BMP4, Wnt*, and *Activin-Nodal*. BMPs are required for the dorsoventral axes of the embryo. Other factors, such as *VG1*, *Nodal*, *Wnt8C*, and *Fgf8*, are required for streak formation. Left–right symmetry and anteroposterior (cranio–caudal) patterning during gastrulation are performed by the expression of the *Nodal* gene, which is mediated by the *SHH* gene [25,30]. The *SHH* gene is a crucial component of craniofacial development, and the loss of *SHH* signaling in the initial stages of neural plate patterning has a substantial effect on craniofacial morphogenesis [56,57].

The important role of the *Nodal* and *BMP* genes in the establishment of the main axes of the embryos was demonstrated by Xu et al., who managed to induce a secondary axis by injecting mRNAs of *Nodal* and *BMP* in zebrafish [58]. Further, *Wnt* mRNA injection induced complete duplication of the axis [25,59]. Because the mouse embryo axis of polarity formation occurs before embryonic genome activation, it is assumed that maternal morphogenes can be implicated in modifications of the polarity, which can lead to embryo duplications [25,60]. Related to this hypothesis, over-aged oocytes can be considered a possible trigger for duplication in cattle [27].

As in all congenital malformations, along with the genetic factors described above, environmental factors can play a role in triggering the congenital malformations, such as (e.g., toxic agents; plants—the intake of Lupinus spp., Conium spp., Mimosa spp., or Nicotiana spp., infectious agents, and exogenous hormone treatments) [2,52,61]. In fish, a possible cause of duplication could be water pollution and water salinity, based on the case of conjoined twins in *Gambusia holbrooki* fish connected at their heads identified in Rambla Salada, a hypersaline stream from the Segura River basin, Spain [20]. It is very important that each case of rare, conjoined twins be reported by breeders and veterinarians with as much data as possible (e.g., the genealogical tree of the affected, the genetic background, animal conditions, nutrition, infectious diseases, and toxic plant ingestion) in order to be able to identify the possible cause. The negative impact of such cases on livestock farmers can be seen as economic losses. Expanding the knowledge in this field can help reduce the incidence of such anomalies.

## 5. Conclusions

Congenital malformations are important pathological conditions in all species. Despite the fascination with conjoined twins throughout history, few cases of swine conjoined twins have been reported in the literature to date. These cases are unique due to their particular anatomical findings and their frequent syndromic evolution. To our knowledge, this article is the first scientific report of conjoined twin piglets from Romania.

New statistical data on conjoined twins do not exist, and we hope that this detailed report will contribute to the knowledge of the incidence of conjoined twins in swine. Nonetheless, the exact mechanism of primitive streak duplication with or without laterality defects remains unknown. Thus, future approaches should focus on genetic and molecular processes.

## Figures and Tables

**Table 1:** The main body measurements of the conjoined twins, case No. 1.

Body Measurements	Left Twin	Right Twin
Crown–rump length	265 mm	263 mm
Head–tail length	330 mm	332 mm
Chest width	241 mm	
Total newborn weight	1058 g	

**Table 2:** The main body measurements of the conjoined piglets, case No. 2.

Body Measurements	Left Twin	Right Twin
Crown–rump length	298 mm	297 mm
Head–tail length	375 mm	371 mm
Chest width	290 mm	
Total newborn weight	1497 g	

## Data Availability

Data are contained within the article.
